# The risk of surgery-related pressure ulcer in diabetics: A systematic review and meta-analysis

**DOI:** 10.1016/j.amsu.2021.102336

**Published:** 2021-04-17

**Authors:** Ebrahim Nasiri, Aghil Mollaei, Moslem Birami, Mojgan Lotfi, Mohammad Hossein Rafiei

**Affiliations:** a Department of Anesthesiology and Operating Room, Faculty of Allied Medical Sciences, Mazandaran University of Medical Sciences, Sari, Iran; b Faculty of Health, Student Research Committee, Mazandaran University of Medical Sciences, Sari, Iran; c School of Allied Medical Sciences, Student Research Committee, Mazandaran University of Medical Sciences, Sari, Iran; d Department of Medical Surgical Nursing, Faculty of Nursing and Midwifery, Tabriz University of Medical Sciences, Tabriz, Iran

**Keywords:** Diabetes mellitus, Operating rooms, Risk factors, Pressure ulcers

## Abstract

**Background and objective:**

Postoperative pressure ulcers are known as the most important quality indicators of intraoperative care that create critical and costly complications during hospital care. Accordingly, this study was performed to determine the risk factor for diabetes in postoperative pressure ulcers.

**Materials and methods:**

The present study is a systematic review of PubMed, Scopus and the Web of Science databases with using standardized keywords of the performed English language articles between Jan 2010 to Jan 2020. The articles were searched independently by two related researchers to avoid possible biases. Then, all collected articles were reviewed, and articles with inclusion criteria were evaluated using a data collection table. It should be noted that the data were analyzed using STATA software version 11.1.

**Results:**

Overall, the results showed that 19724 patients were identified from 15 studies conducted in Asia (six), the America (four), Europe (four), and Australia (one) from 1989 to 2019. The results showed that patients with diabetes were more likely to experience surgery-related pressure ulcers than patients without diabetes (The odds ratio of 1.52; the 95% confidence interval: 1.25–1.85).

**Conclusion:**

In general, patients with diabetes increased the risk of surgery-related pressure ulcers about 1.5 times more than others. Accordingly, the reduction of surgery-induced pressure ulcers should be more extensively considered in patients with diabetes.

## Highlights

**What is already known about the topic?**

• Diabetes as a preoperative comorbidity should be added to risk assessment instruments for perioperative pressure ulcers, which may increase the accuracy of the prediction.
• The excess risk of pressure ulcers associated with pre-existing diabetes was significantly higher in patients undergoing surgery, specifically in patients receiving cardiac surgery.

**What this paper adds**.

• Diabetes increases the risk of surgery-related pressure ulcers about 1.5 times. Therefore, it is necessary to provide Planned Cares to prevent, overcome, and decrease surgery-related pressure ulcers in patients with diabetes.
• Previous meta-analysis reported that the risk of surgery-related pressure ulcers in diabetic patients was higher than non-diabetic patients in cardiac surgeries, while evidence for this claim was not observed in this study. Considering the long duration of liver resection surgery, the risk of pressure ulcers is higher than heart surgery
• It is advised that standard wound measuring tools will apply for measuring wounds in the next prospective studies. It is also better to evaluate pressure ulcers at a specific time after surgery toward more carefully investigate the issue.

## 1 Introduction

Despite notable improvements in patients' health, surgery-related pressure ulcers have remained major health problems and critical challenges for healthcare providers, identifying factors affecting pressure ulcers in patients has been considered as a key factor for care teams [1]. Pressure ulcers are localized injuries to the skin and underlying tissues usually create due to the pressure, or pressure in combination with shear on bony prominences [2]. It has pronounced that pressure ulcer is one of the most common factors affecting patients' prolonged hospitalization after surgery with an incidence of 3.4–66% [3]. Pressure ulcers can be associated with numerous complications, such as pain, reoperation, scar treatment, increased hospitalization, treatment expenses, and other expenses. Furthermore, some clinical reports have indicated that pressure ulcers-induced deep tissue injuries resulted in sepsis, renal failures, and death [2]. Also, operating room treatment costs for pressure ulcers were estimated equal to 750 million to $1.5 billion by previous studies. Adequate knowledge and understanding of the care teams about pressure ulcers and their dangerous consequences can be considered as a basic approach to prevent their incidence [4]. Accordingly, some pressure ulcers risk factors, such as anesthetic-induced immobility, hemodynamic fluctuations, hypothermia during surgery, hypotension, disruptive factors of tissues' tolerance (friction, pressure, moisture), use of surgical tools, and patient position occur during surgery [3,5], increase the risk of developing pressure ulcers. Also, other factors including senility, diabetes, smoking, peripheral vascular disease, malnutrition, low weight, and Hypoalbuminemia, and Hypoproteinemia are contributed to the occurrence of these ulcers [[5], [6], [7]]. Among these contribution factors, diabetes is thought by some clinicians to be the most important factor as it is one of the morbidity factors in patients undergoing surgery which can reduce blood flow in epidermal layers then damages the vascular structure, therefore increase the risk of occurrence pressure ulcer [7,8]. These published reports have varied by incidence, type of surgery, and risk factors, among other reasons [3].

Some meta-analysis studies have only investigated published data related to pressure ulcers as of 2013 [3,5,9]. Previous studies have demonstrated significant associations between diabetes and surgery-related pressure ulcers but in the previous three Meta-analyses studies, there is no mention of wound measuring tools. Therefore, these results did not have good homogeneities, and hence, the report of their results is discussed. On the other hand, with advances related to patient care during surgery and research development in recent years, the use of the results of newer studies can be useful in identifying and preventing complications or healing patients. Therefore, the present study aimed to determine the role of diabetes risk factors on the incidence of surgery-related pressure ulcers.

## 2 Methods

**Fig. 1:**
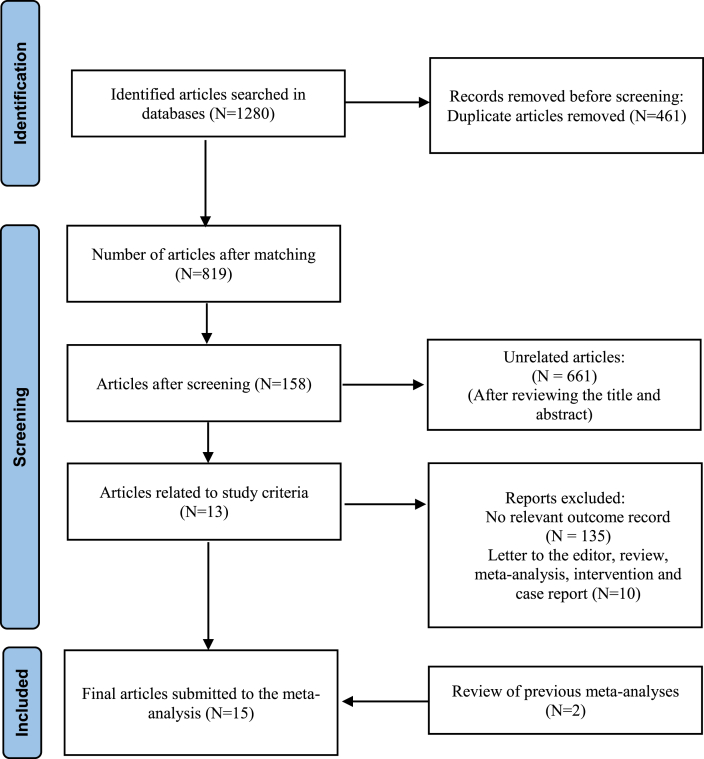
PRISMA flow chart showing selection steps studies for systematic review and meta-analysis.

This review has been reported in line with PRISMA (Preferred Reporting Items for Systematic Reviews and Meta-Analyses) (Fig. 1). And AMSTAR-2 (Assessing the methodological quality of systematic reviews) Guidelines. It was prospectively registered on PROSPERO (ID: CRD42021236820).

### 2.1 Search strategy

The present study is a systematic review study in which was used reviewing documents and available references to determine the effects of the risk factor for diabetes mellitus on the incidence of surgery-related pressure ulcers. Accordingly, articles published in the English language from the PubMed, Scopus, Web of Science databases between Jan 2010 to Jan 2020 were employed to find subjects related to the present study. In general, the desired articles were identified using PECO formulation guidance and systematic search for keywords of pressure ulcer, pressure wound, pressure injury, bedsore and surgery, operation room, surgical patient, preoperative, perioperative, Postoperative, intraoperative, diabetes, diabetes mellitus, and hyperglycemia. Also, OR and AND operators and other available domains of Advanced Search were employed for all databases toward identifying desirable articles. It should be noted that all steps were performed by two expert researchers to avoid the risks of possible bias. Eventually, the obtained data transferred to the Endnote software.

In the first step, the search was performed based on the title and abstracts by two researchers separately. When trying to choose the right topic for our research was not successful based on the available data, the full texts of all articles were then employed for further evaluations by research evaluators, and finally, the appropriate topic was suggested for the present study.

### 2.2 Inclusion and exclusion criteria

Various inclusion criteria, such as initial English language studies, original epidemiological studies (cohort or case-control studies), studies related to diabetes mellitus in patients with the existence of the main dependent variable of pressure ulcers, the association of diabetes with risk of surgery-related pressure ulcers in patients, and having a minimum score of STROBE checklist, were considered in this study. On the other hand, letters to editors, review articles, case studies, and failure to review the main issue were identified as exclusion criteria.

### 2.3 Quality appraisal

Relevant studies were independently evaluated by two researchers using the Strengthening the Reporting of Observational Studies (STROB) in Epidemiology Statement [10]. This Statement consists of a checklist of 22 items in which evaluate various aspects of the methodology, such as sampling methods, measurement of variables, statistical analysis, and study objectives. Also, the minimum and maximum achievable scores inserted in this checklist have been considered equal to 15.5 and 44, respectively. Accordingly, studies with scores of more than 15.5 were included in the present study. In the next step, data were extracted from qualified studies using the data extraction form.

### 2.4 Data collection and statistical analysis

The data extraction form includes the first author surname, geographical location of the study area, date published, study type, study periods, sample size, sex, average age, type of surgery, diabetes, and the incidence of postoperative pressure ulcers. Finally, the obtained data were analyzed using STATA software. We retrieved or calculated the OR estimates with a 95% CI from the baseline form. Statistical heterogeneity was explored by chi-square and Inconsistency (I^2^) statistics; an I^2^ value of 50% or more represented substantial heterogeneity. And Potential publication bias was evaluated by the funnel plot. Funnel plots are a visual tool for investigating publication and other bias in meta-analysis.

## 3 Results

In the primary search, 1280 articles were founded by two researchers, but their numbers were limited to 819 articles after removing the duplicate cases. Then, the mentioned articles were screened based on the title and abstract items. Accordingly, 158 full-text articles were identified as articles related to the present study, while 661 articles were recognized as irrelevant articles. On the other hand, it was performed critically evaluating available complete studies and excluded 145 studies that did not meet the inclusion criteria. It should be pointed out that 13 articles were qualified, and two articles were added manually based on previous meta-analysis references. Finally, the present study was started with 15 studies following a qualitative evaluation stage of the research.

In general, 19724 patients met the inclusion criteria in accepted studies so that 2821 were diagnosed as diabetic patients. Samples' size ranged from 102 to 5966 patients. All entered studies were observational in which seven articles were retrospective studies (case-control), and eight studies were prospective cohorts. According to the geographical location of the study area, available papers were also divided into four categories of conducted studies in Asia (six), America (four), Europe (four), and Australia (one) from 1989 to 2019 (the period was not defined in one study). The researches quality for meta-analysis, in terms of the score of the STROBE checklist and other information related to the accepted studies, is presented in Table 1.

**Table 1:** Information extracted from studies entered in meta-analysis, based on inclusion and exclusion criteria (published from 2010 to 2020).

Author	Country	Year	Study type	Study interval	Sample size	Sex	Age	Surgery type	Odd ratio	95% confidence interval	P-value	Wound assessment tools	Wound stage	Wound assessment time(day)	STROB score
Aloweni et al. [11]	Singapore	2019	Retrospective cohort	2015–2016	269(DM = 69)	Male:141 Female:128	63	All surgery	1.63	0.92–2.92	P < 0.09	National Pressure Ulcer Advisory Panel	All stage	–	19.5
Hong-Lin et al. [12]	China	2019	Retrospective cohort	2015–2016	128(DM = 15)	–	PU mean:62.1 Non PU mean: 61.1	Liver resection	2.11	0.84–5.28	P < 0.19	National Pressure Ulcer Advisory Panel	1	1–3	17
Celik et al. [4]	Turkey	2019	prospective cohort	2015–2016	151(DM = 38)	Male:76 Female:75	PU mean:58.26 Non PU mean: 55.15	neurosurgery, Abdominal, Thoracic and cardiovascular	0.95	0.45–2.01	P < 0.89	National Pressure Ulcer Advisory Panel	1 and 2	0–3	18
Gao et al. [13]	China	2018	prospective cohort	2015–2016	194(DM = 38)	Male:987 Female:953	51.03	neurosurgery, orthopedic, cardiac	0.4	0.05–3.09	P < 0.73	A new and relatively reliable assessment model for IAPU	–	–	16
Lu et al. [14]	China	2017	prospective cohort	2015	149(DM = 32)	Male:79 Female:70	PU mean: 54.7 Non PU mean:48.2	cardiovascular	1.22	0.97–1.56	P < 0.15	A new nomogram score for predicting SRPU in Cardiovascular surgical patients.	1 and 2	–	17
Yoshimura et al. [15]	Japan	2016	Retrospective cohort	2010–2012	277(DM = 9)	Male:112 Female:165	PU mean:45.5 Non PU mean:45.15	brain tumor resection- vascular surgery	1.31	0.12–8.53	P < 0.97	Japanese Ohura-Hotta (OH) scale	1 and 2	1	19
Webester et al. [16]	Australia	2015	prospective cohort	2013	534(DM = 6)	Male:305 Female:299	PU mean:75.17 Non PU mean:53.01	All surgery	2.39	0.43–12.91	P < 0.27	National Pressure Ulcer Advisory Panel	1 and 2	–	15.5
O'Brien et al. [6]	America	2014	Retrospective cohort	2008–2009	2695(DM = 544)	Male:1684 Female:1011	PU mean:61.7 Non PU mean:58.5	All surgery	1.42	1.07–1.88	P < 0.02	National Pressure Ulcer Advisory Panel	2, 3 and 4	–	16.5
Zambonato et al. [17]	Brazil	2013	Retrospective cohort	2005–2006	1503(DM = 243)	Male:711 Female:792	PU mean:58.8 Non PU mean:55.5	All surgery	3.13	1.42–6.92	P < 0.01	Norton Scale (NS)	–	–	18
Ekstroma et al. [18]	Sweden	2013	prospective cohort	–	2133(DM = 234)	Male:585 Female:1548	DM: 82 non-DM: 81	Hip fractures	0.95	0.72–1.25	–	–	–	–	19
Bulfone et al. [19]	Italy	2011	prospective cohort	2009	102(DM = 14)	Male:63 Female:39	62.3	Neurosurgery, cardiac, general, Plastic Surgery	2.2	1.20–4.03	–	National Pressure Ulcer Advisory Panel	1 and 2	0–6	16.5
Tschannen et al. [20]	America	2012	Retrospective cohort	2007–2009	3225(DM = 736)	Male:1910 Female:1315	PU mean:61.7 Non PU mean:58.5	All surgery	1.49	1.14–1.95	P < 0.00	National Pressure Ulcer Advisory Panel	1	–	19
Norris et al. [21]	England	2011	prospective cohort	1989–2008	5966(DM = 477)	Male:1400 Female:4566	DM: 80 non-DM: 73	Hip fractures	2.29	1.60–3.27	–	–	–	–	17
Aragón et al. [22]	Spain	2010	Retrospective cohort	1998–2008	277(DM = 221)	Male:180 Female:103	DM: 78 non-DM: 73	Amputation	1.12	0.23–5.41	P < 0.88	–	–	–	17
Slowikowsk et al. [23]	America	2010	prospective cohort	2005–2008	277(DM = 87)	Male:208 Female:161	58.3 ± 19.3	All surgery	1.93	1.25–1.85	P < 0.01	Pressure Ulcer Risk Assessment (SPURA) scale	–	–	16

Abbreviations: PU= Pressure Ulcer, DM = Diabetic Mellitus.

A preliminary meta-analysis was performed using 15 studies that met the inclusion criteria in the final analysis of the present study. The odds ratio for studies focusing on the association of diabetes with the incidence of pressure ulcers during the surgical process was assessed equal to 1.52 and confidence interval 95% (CI95%) equal to 1.25–1.85. Plus, the index of I^2^ and Q test were calculated for studies with heterogeneity examination.

**Fig. 2:**
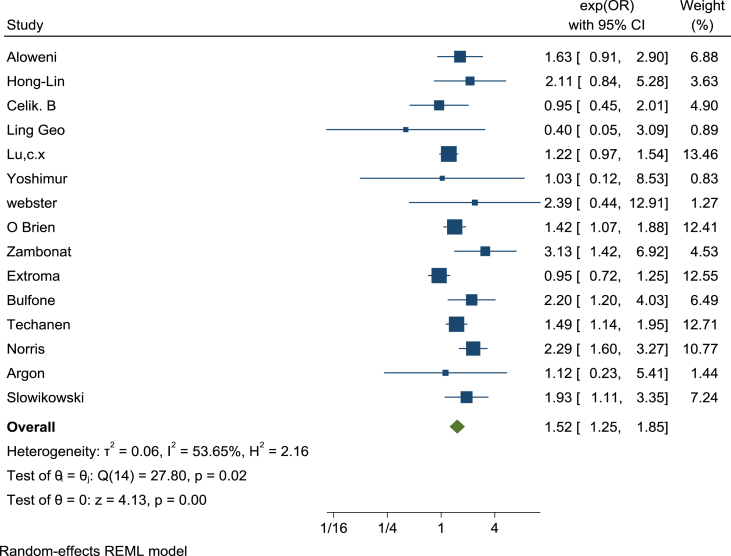
Forrest Plot The rate of pressure ulcer in surgical patients with diabetes versus non-diabetic patients based on the random effect model, the midpoint of each segment shows the odds ratio and the length of each segment shows a 95% confidence interval in each study. The rhombus sign is the odds ratio in all studies.

Furthermore, considering the heterogeneity of the studies, the Random Effects Model was used to combine the results of the existing studies (Fig. 2).

Subgroup analysis approaches were used to identify possible reasons for heterogeneity in the mentioned studies. Subgroup analysis based on the study type revealed that the odds ratio in retrospective and prospective studies was evaluated equally to 1.51 (CI95% = 1.42–2.06 and p < 0.6) and 1.44 (CI95% = 0.99–2.21 and p < 0.00), respectively. On the other hand, despite the heterogeneity in prospective studies (I^2^ = 64%), there was no heterogeneity in retrospective studies (I^2^ = 00%).

Regarding the further investigation of the heterogeneity among studies, subgroup analysis was performed based on the study location in four continents of USA, Europe, Asia, and Australia, which observed significant heterogeneity in studies conducted in Europe (I^2^ = 77%). Subgroup analysis did not reveal any heterogeneity between groups concerning the type of surgery except two performed studies of hip fracture surgery (I^2^ = 93%). Plus, the subgroups' analysis illustrated that two studies of Extroma and Norris were identified as heterogeneous factors for three items of retrospective and prospective study, study area, and type of surgeries (Table 2).

**Table 2:** Analysis of subgroups, the relationship between diabetes and the risk of pressure ulcers in patients undergoing surgery.

Variable	OR	CI95%	Heterogenic (I^2)	P-value	Studies number	P-value*
total	1.52	(1.25–1.85)	53.65	0.02	15	
Study type						0.74
Prospective cohort	1.51	(1.24–1.58)	0.00	0.64	7	
Retrospective cohort	1.44	(1.05–1.99)	68.67	0.00	8	
Surgery type						0.4
Liver resection	2.11	(0.84–5.28)	.		1	
neurosurgery, Abdominal, Thoracic and cardiovascular	0.95	(0.45–5.01)	.		1	
neurosurgery, orthopedic, cardiac	0.4	(0.05–3.09)	.		1	
cardiac	1.22	(0.96–1.54)	.		1	
brain tumor resection- vascular surgery	1.03	(0.12–8.53)	.		1	
All surgery	1.57	(1.33–1.86)	0.00	0.5	6	
Hip fractures	1.41	(0.61–3.47)	93.15	0.00	2	
Amputation	1.12	(0.23–5.41)	.		1	
Region						0.4
America	1.55	(1.3–1.86)	0.00	0.25	4	
Asia	1.25	(1.03–1.54)	0.00	0.54	6	
Europa	1.43	(0.7–2.92)	77.88	0.00	4	
Australia	2.39	(0.44–12.9)	.	.	1	
Study interval						0.02
After 2010	1.27	(1.04–1.55)	0.00	0.62	7	
Befor 2010	1.78	(1.44–2.22)	36.53	0.19	7	
Sample size						0.92
<1000	1.49	(1.17–1.9)	19.90	0.49	9	
>1000	1.52	(1.07–2.16)	78.07	0.00	6	
Mean age pressure ulser						0.68
<60	1.38	(1.1–1.74)	24.48	0.16	5	
>60	1.48	(1.14–1.94)	0.00	0.67	3	
Female percentage						0.56
<50	1.41	(1.23–1.61)	0.00	0.51	10	
>50	1.71	(0.9–3.23)	81.76	0.00	4	

P-value: p-value in the test for differences between groups *.

In the sensitivity analysis, the Extroma study was excluded by the leave-one-out technique, and the results related to the rest of the studies had homogeneous positions (OR = 1.53; CI95% = 1.35–1.73; I^2^ = 28%).

**Fig. 3:**
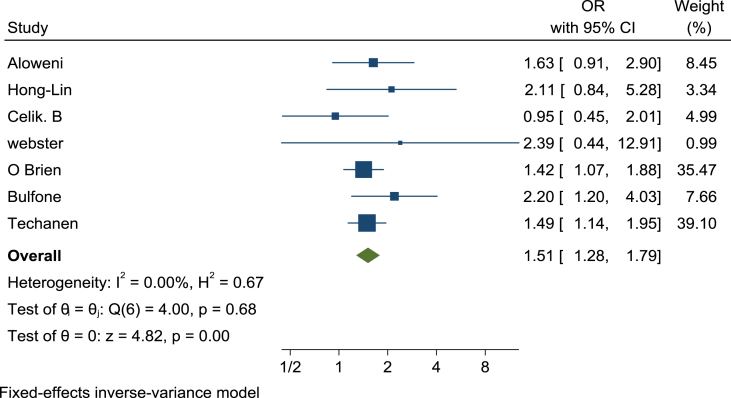
The rate of pressure ulcers in surgical patients with diabetes versus non-diabetic patients based on the fixed model, in studies that have used the tools of “National Pressure Ulcer Advisory Panel” to evaluate wounds.

Seven numbers of the 15 entered studies in the present study were jointly employed the National Pressure Ulcer Advisory Panel tool to evaluate surgical-related pressure ulcers, but the rest studies were used different tools. Subgroup analysis performed in these seven studies indicated that the odds ratio for surgery-related pressure ulcers in diabetic patients (CI95% = 1.28–1.79; I^2^ = 00%) was 1.51 times higher than non-diabetic patients (used fixed-effect model) (Fig. 3).

Data extracted from 15 studies have shown that in the majority of them, pressure ulcers occurring during surgery, were stage 1 or 2 and A small number of studies mentioned the exact time of wound evaluation after surgery(Table 1).

**Fig. 4:**
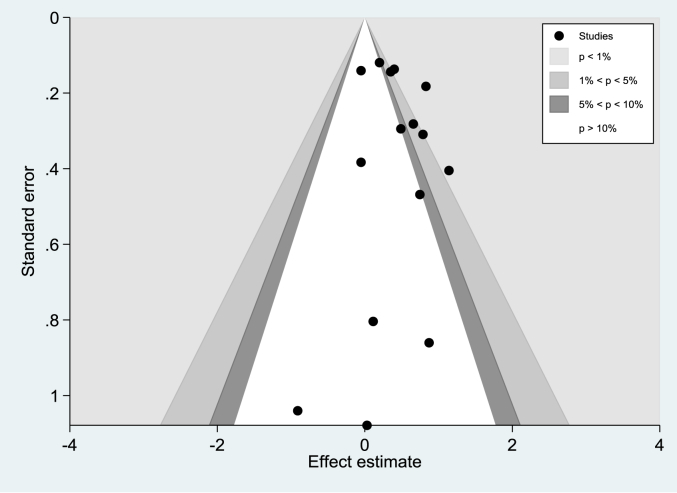
Publication bias detected by funnel plot, SE: Standard Error.

The symmetry lines in Fig. 4 indicate the lack of bias for the published results. It was also supported by the egger's test with a p-value of 0.815.

## 4 Discussion

The results of our systematic review and meta-analysis, which summarized the results of 15 observational studies with 19724 surgical patients, confirmed the association between diabetes mellitus and risk factors for the occurrence of intraoperative pressure ulcers in patients. Evidence showed that the risk factor for surgery-related pressure ulcers in diabetes patients (CI95%, 1.25–1.85) was 1.52 times higher than non-diabetic patients.

The obtained results of this study support the primary conclusions of the three previously published meta-analyses. Liu reported that the odds ratio for surgery-related pressure ulcers in diabetic patients with CI95% of 1.62–2.84 equal to 2.15 times higher than non-diabetic patients [9]. In a similar study, Kang et al. (2015) also displayed the results in agreement with the previous research (OR = 1.74; CI95% = 1.40–2.15) [3]. Furthermore, Liang et al. (2017) reported significant results for the risk of diabetes toward the incidence of pressure ulcers after surgery (RR = 1.77; CI95% = 1.45–2.16) [7].

Although our findings were significantly in line with the results of the three previous studies, the impact values reported in previous studies were slightly higher than the present study. The above results may be due to the preventive measures taken to control patients' pressure ulcers in recent years.

One of the objections to the previous three meta-analytic approaches is not attention to wound measuring tools in the studies. Among the analyzed studies, seven studies employed similar tools, and the rest of the studies used different tools in evaluating surgery-related pressure ulcers. Overall, in the previous seven studies, the National Pressure Ulcer Advisory Panel tool was applied as the standard tool. The pressure ulcer classifications presented by the National Pressure Ulcer Advisory Panel have been focused clinically on ulcer features in four stages. In stage 1, an arrhythmia happens at the site of pressure that does not whiten by finger pressure, which is a sign of pressure ulcers in the future. Symptoms of bruising, warmth, and stiffness may appear at the site of the pressure. Stage 2, shows a decreased thickness for the skin. The ulcer is superficial and presents clinically as an abrasion, blister, or shallow crater. Also, it should be noted that pressure ulcers are usually painful at this stage. Stage 3 involves expanding down the skin thickness to the fascia. In stage 3, pressure ulcer wounds developed into the deep of fascia and sometimes develop to surrounding tissues. Wound healing at this stage takes months. In stage 4, the full-thickness of the tissue is eliminated, which is associated with tissue necrosis, damage to the muscles, bones, and related structures, tendons, and joint capsules. Recovery at this stage could take months up to a year or even longer [11].

In the performed meta-analysis by Kang et al. (2015), it was reported that the risk of surgery-related pressure ulcers in diabetic patients was higher than non-diabetic patients in cardiac surgeries, while evidence for this claim was not observed in this study. Considering the long duration of liver resection surgery, the risk of pressure ulcers is higher than heart surgery. Accordingly, it can be concluded that the incidence of pressure ulcers has no significant relationship with the type of surgery (or cardiac surgery), but the time of surgery can affect this process [3].

In the present study, since all published articles from 2013 to 2020 were collected, summarized, and analyzed, it can be stated that the previous meta-analyses were updated and upgraded. In addition, this study tried to describe and resolve the limitations mentioned in previous meta-analyses, such as the use of limited databases, highly sensitive search, search with different keywords, and ignoring the tools used to evaluate pressure ulcers.

### 4.1 Research limitations

Limitations of the present study included only evaluating articles published in the English language. Also, in the articles included in the present study, there was no evidence of the meta-analysis of pressure ulcers at a certain time after surgery.

## 5 Conclusion

Our meta-analysis findings showed that diabetes increases the risk of surgery-related pressure ulcers about 1.5 times. Therefore, it is necessary to provide Planned Cares to prevent, overcome, and decrease surgery-related pressure ulcers in patients with diabetes. However, it is advised that standard wound measuring tools will apply for measuring wounds in the next prospective studies. It is also better to evaluate pressure ulcers at a specific time after surgery toward more carefully investigate the issue.

## Provenance and peer review

Not commissioned, externally peer reviewed.

## Ethical approval

IR.MAZUMS.REC.1399.931.

## Sources of funding

No.

## Author contribution

Mohammad Hossein Rafiei and Ebrahim nasiri did overall supervision, material provision, study conception. And Mohammad Hossein Rafiei and Moslem Birami did search and data accumulation. Aghil Mollai did statistical analysis, data provision. Mohammad Hossein Rafiei did data provision, manuscript preparation. Ebrahim Nasiri and Mojgan Lotfi did manuscript preparation, final edit, study conception.

## Registration of research studies

1. Name of the registry: PROSPERO.

2. International prospective register of systematic reviews.

3. Unique Identifying number or registration ID: CRD42021236820.

4. Hyperlink to your specific registration (must be publicly accessible and will be checkedhttps://www.crd.york.ac.uk/prospero/#loginpage.

Email Address: Hosein1373333@gmail.com.

Password: 1361439858mhR

## Guarantor

Mohammad Hossein Rafiei and Ebrahim nasiri.

## Declaration of competing interest

All authors have no conflict of interest to report.
